# Childhood Family Environment and Osteoporosis in a Population‐Based Cohort Study of Middle‐to Older‐Age Americans

**DOI:** 10.1002/jbm4.10735

**Published:** 2023-03-26

**Authors:** Margaret Gough Courtney, Josephine Roberts, Yadira Quintero, K. Godde

**Affiliations:** ^1^ Department of Sociology/Anthropology University of La Verne La Verne California USA

**Keywords:** AGING, GENERAL POPULATION STUDIES, OSTEOPOROSIS, PRACTICE/POLICY‐RELATED ISSUES, STATISTICAL METHODS

## Abstract

Demographic and early‐life socioeconomic and parental investment factors may influence later‐life health and development of chronic and progressive diseases, including osteoporosis, a costly condition common among women. The “long arm of childhood” literature links negative early‐life exposures to lower socioeconomic attainment and worse adult health. We build on a small literature linking childhood socioeconomic status (SES) and bone health, providing evidence of whether associations exist between lower childhood SES and maternal investment and higher risk of osteoporosis diagnosis. We further examine whether persons identifying with non‐White racial/ethnic groups experience underdiagnosis. Data from the nationally representative, population‐based cohort Health and Retirement Study (*N* = 5,490–11,819) were analyzed for participants ages 50–90 to assess these relationships. Using a machine learning algorithm, we estimated seven survey‐weighted logit models. Greater maternal investment was linked to lower odds of osteoporosis diagnosis (odds ratio [OR] = 0.80, 95% confidence interval [CI] = 0.69, 0.92), but childhood SES was not (OR = 1.03, 95% CI = 0.94, 1.13). Identifying as Black/African American (OR = 0.56, 95% CI = 0.40, 0.80) was associated with lower odds, and identifying as female (OR = 7.22, 95% CI = 5.54, 9.40) produced higher odds of diagnosis. There were differences in diagnosis across intersectional racial/ethnic and sex identities, after accounting for having a bone density scan, and a model predicting bone density scan receipt demonstrated unequal screening across groups. Greater maternal investment was linked to lower odds of osteoporosis diagnosis, likely reflecting links to life‐course accumulation of human capital and childhood nutrition. There is some evidence of underdiagnosis related to bone density scan access. Yet results demonstrated a limited role for the long arm of childhood in later‐life osteoporosis diagnosis. Findings suggest that (1) clinicians should consider life‐course context when assessing osteoporosis risk and (2) diversity, equity, and inclusivity training for clinicians could improve health equity. © 2023 The Authors. *JBMR Plus* published by Wiley Periodicals LLC on behalf of American Society for Bone and Mineral Research.

## Introduction

An extensive literature on the “long arm of childhood” links early‐life exposures, especially childhood economic resources, to adult health outcomes.^(^ ^1^, ^2^, ^3^ ^)^ Numerous studies link childhood socioeconomic status (SES) with chronic^(^ ^4^ ^)^ and progressive^(^ ^1^ ^)^ health conditions in later life. However, prior research was limited in what types of health outcomes could be studied. For example, to date it is not well understood how early‐life experiences or childhood SES might affect risk of osteoporosis, which is a common, and costly, progressive disease^(^ ^5^ ^)^ that is one of the 20 health conditions targeted for improvement in the Healthy People 2030 objectives (https://health.gov/healthypeople/objectives-and-data/browse-objectives), that most often affects women,^(^ ^6^ ^)^ and that leads to reduced quality of life.^(^ ^7^ ^)^ We build on the few studies that examine this relationship,^(^ ^8^, ^9^ ^)^ asking how childhood SES and maternal investment link to later osteoporosis diagnosis among middle‐ and older‐aged adults in the United States (from the Health and Retirement Study [HRS]), accounting for key demographic and other variables that may serve as confounders or mediators. Because research linking parental factors to osteoporosis is so limited, we conducted an analysis of association to establish a foundation for understanding plausible relationships that could be tested in future causal studies.

### Childhood SES, maternal investment, and the long arm of childhood

Numerous components contribute to SES, including education (and parental education for children), income, and wealth. Parental education further links with health; individuals whose parents have/had lower education levels have worse physical outcomes than those whose parents have/had higher education if the individuals themselves are also poorly educated.^(^ ^10^ ^)^ However, obtaining at least a college education appears to mitigate the effects of low parental education.^(^ ^10^ ^)^

**Fig. 1:**
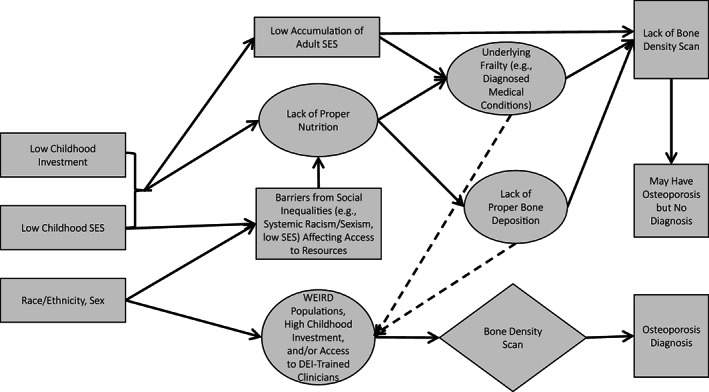
Conceptual model. WEIRD = White, Educated, Industrialized, Rich, and Democratic.

Childhood SES has numerous long‐term effects, and several mechanisms for this relationship have been posited. We consider two main theoretical pathways. First, childhood SES affects SES accumulation as an adult, through educational attainment, intergenerational transmission of wealth, and related factors, and adult SES strongly relates to health outcomes, including risk of osteoporosis diagnosis. Link and Phelan describe (adult) SES as a fundamental cause of health inequities.^(^ ^11^ ^)^ Thus, if childhood SES and parental investments (discussed in what follows) affect SES accumulation in adulthood, this could influence later‐life health and perhaps the likelihood of being diagnosed with certain conditions, like osteoporosis. This is because: (1) greater education and/or other measures of SES in adulthood may reduce the likelihood of developing a disease and (2) greater education, income, and other forms of flexible resources improve individuals' abilities to receive diagnoses and treatment for a disease. Second, low SES during childhood, and possibly low maternal investment, may result in nutritional deficiencies that have long‐term effects on health, including bone density through improper bone deposition.^(^ ^12^, ^13^, ^14^, ^15^ ^)^ Although improper bone deposition is likely to lead to osteoporosis, the disadvantaged backgrounds of many of these individuals reduce the likelihood of receiving a diagnosis of osteoporosis because diagnosis requires access to a bone mineral density (BMD) scan. However, we argue that access to a more privileged position (e.g., those in White, Educated, Industrialized, Rich, and Democratic [WEIRD] populations) or to a clinician trained in issues of diversity, equity, and inclusion (DEI) would make these individuals more likely to receive a bone scan and subsequent diagnosis. We demonstrate these pathways in Figure 1.

Maternal investment is one potentially novel metric of investment that complements childhood SES. In this study, maternal investment roughly correlates with Western ideas of “good mothering” because of the construction of available variables. For example, rather than thinking about maternal investment in the sense of prenatal investments by mothers (e.g., nutritional investments), the focus is on aspects such as the effort mothers put into providing a “good upbringing,” teaching their children about life, and maternal time and attention.^(^ ^16^ ^)^ Maternal investment, as defined in this study, is a form of social capital deriving from the existence and quality of relationships between mothers and their children.^(^ ^16^ ^)^

Maternal investment and parental social capital connect to attainment of adult SES. Dufur et al. show higher parental social capital relates to higher test scores, which are a key predictor of educational attainment.^(^ ^17^ ^)^ Likewise, von Otter and Stenberg find improved academic performance among individuals with a combination of higher familial social capital and good parent–child relationships.^(^ ^18^ ^)^ Like childhood SES, maternal investment may also be connected through nutritional pathways to later‐life health. In developing countries, whose circumstances in some ways reflect those experienced among low‐income U.S. families 50–90 years ago (which coincides with the timeframe the participants in the HRS were undergoing maternal investment), greater maternal social capital, especially cognitive social capital, links to better nutritional status, as proxied by weight for age and height for age.^(^ ^19^, ^20^ ^)^ Research from the United States shows that higher levels of maternal stress relate to higher risk of obesity among food‐secure, low‐income children compared to food‐insecure children,^(^ ^21^ ^)^ suggesting a critical role for maternal circumstances in child nutrition. In contemporary literature, greater maternal investment links to better self‐rated health and fewer depressive symptoms among middle‐ to older‐age adults.^(^ ^16^ ^)^ Recently, a lower hazard of osteoporosis was estimated from greater maternal investment in a Cox regression, suggesting an age‐dependent relationship with later bone health.^(^ ^22^ ^)^

Early‐life experiences, especially those related to SES and parental investment, may be a key factor for later‐life bone health and receiving an osteoporosis diagnosis, the latter of which is known to be inequitably distributed throughout the population and is an important precursor to treatment and avoidance of subsequent fractures that can lead to excess morbidity and mortality. Yet the literature also suggests a number of caveats. Higher childhood SES is associated with higher values of femoral neck strength (reducing osteoporosis risk) for people who identify as White males, but not for those with other identities.^(^ ^9^ ^)^ Relatedly, individuals with greater childhood socioeconomic advantage have higher adult lumbar spine BMD, but there is no relationship with femoral neck BMD.^(^ ^8^ ^)^ These studies did not demonstrate a clear pattern across bone sites and groups, suggesting a need for further investigation.

### Importance of osteoporosis as an outcome of study

Studies of the long arm of childhood for adult health have primarily focused on acute health problems (e.g., heart attack),^(^ ^23^ ^)^ chronic conditions (e.g., hypertension),^(^ ^4^, ^23^ ^)^ and mortality.^(^ ^2^ ^)^ These outcomes are important contributors to morbidity and mortality in the United States. However, it is useful to consider other chronic and progressive conditions that may be a major source of disability. Osteoporosis is one such condition. Osteoporosis is a progressive condition that occurs when bone tissue, structure, and strength begin to deteriorate.^(^ ^24^ ^)^ It is a costly condition, with the bulk of the burden attributable to osteoporotic fractures^(^ ^25^ ^)^; the cost of osteoporotic fractures was estimated to be $57 billion in 2018 in the United States, with $48.8 billion deriving from direct medical costs and the remainder from indirect costs (e.g., related to loss of productivity and caregiving).^(^ ^5^ ^)^ Furthermore, osteoporosis results in significant reductions in quality of life.^(^ ^7^, ^25^ ^)^ As the proportion of the United States population at older ages continues to increase, the osteoporosis‐related burden of morbidity and mortality is likely to increase as well unless there is movement to more effectively prevent, diagnose, and treat osteoporosis.^(^ ^5^ ^)^ Osteoporosis is more common in females than males,^(^ ^6^ ^)^ but males have a higher hazard of mortality after experiencing an osteoporotic fracture.^(^ ^26^ ^)^ Osteoporosis is at least partially preventable and is underdiagnosed, even among those experiencing a fragility fracture.^(^ ^5^, ^27^ ^)^ Thus, it is important to identify risk factors for developing osteoporosis, to understand the factors linked to receiving an osteoporosis diagnosis, and to understand how these are distributed across the population.

### Confounding, mitigating, and enhancing factors

Given the important link between childhood and adulthood SES, and the apparent role that education plays in this linkage, education may mediate between early‐life exposures and later‐life health.^(^ ^28^ ^)^ Conversely, education may be an exposure of its own. Education can be thought of as a flexible resource to be deployed to improve health and well‐being, similar to other forms of SES.^(^ ^29^ ^)^

It is impossible to study the role of early‐life exposures for later‐life health without considering other relevant factors that may relate to life course experiences, including demographic characteristics like respondent sex and race/ethnicity. Race/ethnicity is an important variable for understanding early‐life exposures and later‐life health because structural racism is omnipresent in U.S. society. Race/ethnicity also links to childhood SES through structural racism and plays a role in the achievement of adult SES as well.^(^ ^30^ ^)^ Phelan and Link argue that “racial inequalities in health endure primarily because racism is a fundamental cause of racial differences in SES and because SES is a fundamental cause of health inequalities.”^(^ ^30^ ^)^ Thus, race/ethnicity and SES are highly intertwined for health outcomes. Race/ethnicity is also a key issue in the underdiagnosis of osteoporosis due to significant bias among health care professionals.^(^ ^31^, ^32^ ^)^

Getting an osteoporosis diagnosis in the U.S. health care system can be a challenge, with diagnoses still reflecting race and sex bias due to an outdated understanding of BMD patterning by practitioners, with health equity only achieved through equal access to a bone density scan.^(^ ^33^ ^)^ Thus, SES, race/ethnicity, and sex are all potential barriers to accessing a bone density scan, but having a privileged position across these categories or a DEI‐trained clinician may improve access. In this paper, we explore family/SES environment in childhood and adult demographic characteristics that may influence whether someone receives an osteoporosis diagnosis to provide a foundation for future causal studies, although this paper is an analysis of association and is not causal in nature. Given racial/ethnic bias in the health care system, links between race/ethnicity and SES, the importance of SES and quality of parental relationships for health outcomes and health inequities, evidence of the long arm of childhood exposures, and in keeping with the possible mechanisms discussed previously, we propose the following hypotheses: (1) lower childhood SES will associate with a higher risk of osteoporosis diagnosis in adulthood; (2) separate from childhood SES, lower maternal investment will link to a higher risk of osteoporosis diagnosis; and (3) there will be evidence of underdiagnosis among persons identifying with non‐White racial/ethnic groups, in part through lower access to bone density scans.

## Material and Methods

### Data

**Fig. 2:**
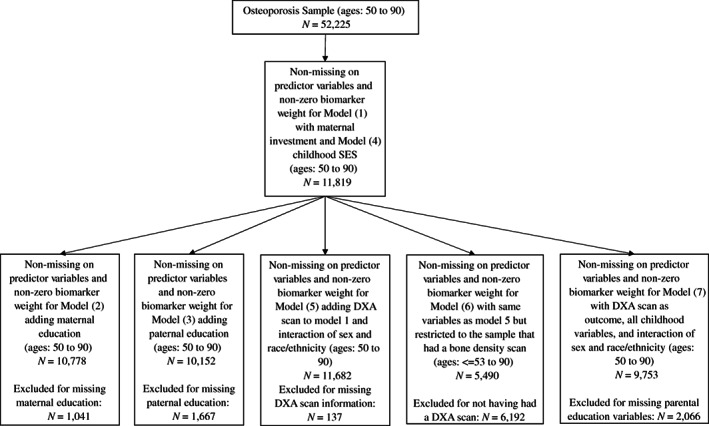
Sample selection flowchart. From top to bottom, the inclusion criteria were having answered the question about osteoporosis diagnosis or having had a bone scan and answering/being measured for each of the covariates. (A complete accounting of how many observations were lost to missing data on each variable is not straightforward. This is because the models were estimated for the subpopulations with non‐missing data on all variables, given the specifications of the survey‐focused estimation program in R. In other words, observations were not dropped sequentially for each variable; rather, a subpopulation was created of all observations without any missing data on model variables.)

Data were obtained from the 2012 to 2016 waves of the HRS^(^ ^34^ ^)^ conducted by the University of Michigan's Institute for Social Research; the response rates and study design are described elsewhere.^(^ ^35^, ^36^ ^)^ We used data from the RAND HRS longitudinal study, which is a cleaned and harmonized version of the core and exit surveys.^(^ ^37^ ^)^ RAND developed the longitudinal study with funding from the National Institute on Aging and the Social Security Administration. The Validated Measures of Childhood Socio‐Economic Status contribution^(^ ^16^ ^)^ was the source of two of our childhood variables: maternal investment and childhood SES. The cross‐wave race/ethnicity study from the HRS data was used in conjunction with core data to create a more complete race/ethnicity variable.^(^ ^38^ ^)^ The Life History Mail Survey component is the source of the data on thyroid disease.^(^ ^39^, ^40^ ^)^ HRS Biomarker data contain several of the biomarkers used to construct our allostatic load variable.^(^ ^41^ ^)^ The inclusion criteria for this research were having responded to the question of whether the respondent had received an osteoporosis diagnosis from a doctor (0 = no, 1 = yes) and having data available for the predictor variables described in what follows. As a result, the sample is limited to the 2012–2016 waves. Participants in the sample (*N* = 5,490–11,819, depending on model; Figure 2) were ages 50–90 for Models 1–6 and nationally representative of the racial/ethnic diversity in the United States. Model 7 differed by age range, which was ≤53–90. We are unable to provide a more definitive lower bound for age in Model 7 as the cell counts below 53 would violate anonymity restrictions for working with these data; for more information see Health and Retirement Study.^(^ ^42^ ^)^ The respondents in this population‐based cohort sample were community dwellers or nursing home residents who were formerly community dwelling. As this is not a randomized clinical trial (RCT), we adhere to the Strengthening the Reporting of Observational Studies in Epidemiology (STROBE) reporting guidelines in this secondary data study (determined exempt by the University of La Verne Institutional Review Board [Protocol No.: 2019‐13‐CAS]). HRS obtained informed consent as part of their data collection efforts, and the protocol is described in the HRS documentation.^(^ ^42^ ^)^

### Variables

The outcome variable for Models 1–6 is the respondent reporting receipt of an osteoporosis diagnosis from a doctor. For Model 7, the outcome variable is whether a respondent reports having ever had a bone density scan. The first exposure variable is maternal investment (continuous index), which measures the quality of the respondents' relationship with their mother, using a Likert scale, and is constructed from items in the core posing the following questions: (1) “How much effort did your mother put into watching over you and making sure you had a good upbringing?” (2) “How much did your mother teach you about life?” (3) “How much time and attention did your mother give you when you needed it?”.^(^ ^16^ ^)^ The second exposure variable, parental education (continuous in years), was enumerated separately by parent in the core. The third exposure variable, childhood SES index (continuous), was developed from core childhood questions regarding social capital, financial capital, and human capital; for detailed information on its construction, see Vable et al.^(^ ^16^ ^)^ All of the childhood variables refer to the time when the respondent was younger than 16. The final exposure was respondent education, also a potential mediator for childhood circumstances, and it was measured categorically (1 = less than high school, 2 = GED, 3 = high school grad, 4 = some college, 5 = college and above). Although we describe these variables as “exposures” in line with STROBE guidelines, we view this paper as explicitly an analysis of association; we do not conduct a causal analysis due to limited existing literature in this area that could inform a causal model.^(^ ^43^ ^)^

Because perception of race/ethnicity (categorical; split across the following identities due to sample size restrictions: 1 = White/European American, 2 = Black/African American, 3 = another race/ethnicity) can influence one's ability to get diagnosed with osteoporosis as a product of systemic racism (e.g., Godde et al.^(^ ^33^ ^)^), it was included. Further, a variable was created combining each identity of sex with each racial/ethnic identity in order to look at intersectionality. Control variables were added, using the factors identified by Gough Courtney et al.,^(^ ^22^ ^)^ who derived an initial set of all possibly related variables for osteoporosis diagnoses in the HRS using theory and the published literature. They applied a filter method (Spearman's correlation) to the initial set of variables to eliminate those that had negligible impact on the outcome variable, that is, a change of 0.01 or less across the covariates, or that were duplicative measures of other variables. After the initial filtering, the change‐in‐estimate method^(^ ^44^ ^)^ was implemented to identify potential confounders. The control predictors were derived from their final set of variables and might have excluded some relating to health, health behaviors, and other demographic factors usually identified as important in the literature because they were not for this particular sample (i.e., from the HRS). Controls included age (measured continuously and transformed with a natural log to address issues of normality), sex (binary; 1 = male, 2 = female), self‐reported health (categorical; 1 = excellent, 2 = very good, 3 = good, 4 = fair, 5 = poor), thyroid disease (binary; 1 = diagnosed, 5 = not diagnosed), weight in kilograms (continuous), and allostatic load [continuous index, derived from McCrory et al.^(^ ^45^ ^)^; for each of the following items, a value of 1 was assigned to individuals with values greater than the 75th percentile, and the 1s were summed: systolic blood pressure (mmHg), diastolic blood pressure (mmHg), pulse (bpm), total cholesterol (mg/dL), high sensitivity‐Creactive protein CRP (mg/dL), A1c (%), Cystatin C (mg/L), and waist circumference (women: >88.9 cm, men: >101.6 cm). Allostatic load was evaluated due to its relationship with stress; environmental factors (e.g., demographic, social, childhood context) can elevate stress, which in turn may trigger an increase in inflammation via the hypothalamic–pituitary–adrenal (HPA) axis.^(^ ^46^ ^)^ Chronic inflammation may lead to increased risk of osteoporosis.^(^ ^47^ ^)^

### Statistical modeling

Four models were developed to describe early‐life experience exposure variables for osteoporosis (Hypotheses 1 and 2). Model 1 incorporates adult demographic measures (age at interview and sex and race/ethnicity identities as an adult) and adds maternal investment to explore its impact on osteoporosis diagnosis. Model 2 includes the addition of mother's education to Model 1, and similarly Model 3 incorporates father's education into Model 1. Finally, Model 4 inserts childhood SES into Model 1.

Models 5–7 test Hypothesis 3 about underdiagnosis and access to bone density scans by using osteoporosis diagnosis as the outcome variable (Models 5–6) or receipt of a bone density scan as the outcome variable (Model 7). For Model 5, we used the variables from Model 1, along with having had a bone density scan and the combined sex and race/ethnicity variable to look at intersectionality. We reran Model 5, restricting the sample to include only people who stated they had a bone density scan (Model 6) and thus dropped the bone density scan variable. Lastly, the predictor variables in Model 7 comprised all predictor variables in this paper, but the outcome variable was whether the respondent had had a bone density scan.

A multivariable logistic regression was run with the binary outcome variable of osteoporosis diagnosis (and having a bone density scan in Model 7). The variance inflation factor (VIF) assessed multicollinearity, and adjusted McFadden's pseudo‐*R* ^2^ and a C statistic assessed model fit and discrimination. Missing data were addressed using listwise deletion; a flowchart of analytic sample sizes is depicted in Figure 2. The HRS uses a complex survey sampling design, which necessitates the use of weights to reduce response bias. We analyzed the data via the survey package^(^ ^48^ ^)^ in R^(^ ^49^ ^)^ using provided biomarker weights and paired with Horvitz‐Thompson standard errors, which increased robustness. This, coupled with a wave variable that was a proxy for the year of data collection across the different types of variables, dealt with repeated measures.

## Results

Descriptive statistics of the analytic sample are found in Tables 1 and 2. Approximately 9% of the sample reported an osteoporosis diagnosis. The sample was slightly more female than male. The mean age was around 70 years old. Across the models approximately 88% of the sample identified as White/European American, 7% as Black/African American, and 4% as another race/ethnicity. The VIFs were all under 10.

**Table 1:** Descriptive Statistics for Samples in Models 1–4

	Models 1 and 4 (*N* = 11,819)	Model 2 (*N* = 10,778)	Model 3 (*N* = 10,152)
Variable	Mean (SE)/Proportion	Mean (SE)/Proportion	Mean (SE)/Proportion
Age	69.81 (0.16)	69.74 (0.17)	69.84 (0.19)
Sex			
Female	0.53	0.54	0.53
Male	0.47	0.46	0.47
Race and ethnicity			
White	0.87	0.88	0.89
Black/African American	0.08	0.07	0.06
Another race/ethnicity	0.05	0.04	0.04
Education level			
Less than high school	0.14	0.11	0.10
GED	0.04	0.04	0.04
High school grad	0.29	0.29	0.29
Some college	0.25	0.26	0.25
College and above	0.28	0.30	0.31
Allostatic load	2.21 (0.02)	2.20 (0.02)	2.18 (0.02)
Weight in kilograms	82.09 (0.26)	82.03 (0.27)	81.97 (0.29)
Thyroid disease (ever)			
No	0.96	0.96	0.96
Yes	0.04	0.04	0.04
Self‐reported health			
Excellent	0.09	0.09	0.10
Very good	0.34	0.35	0.35
Good	0.33	0.33	0.33
Fair	0.18	0.17	0.16
Poor	0.06	0.06	0.05
Maternal investment	−0.09 (0.01)	−0.08 (0.01)	−0.06 (0.01)
Respondent's mother level of education		10.47 (0.07)	
Respondent's father level of education			10.16 (0.08)
Childhood socioeconomic status index	0.27 (0.02)		
Osteoporosis			
No	0.91	0.91	0.91
Yes	0.09	0.09	0.09
Wave	11.90 (0.01)	11.90 (0.01)	11.90 (0.01)
Osteoporosis diagnosis by doctor (2011–12)			
No	0.84	0.84	0.85
Yes	0.16	0.16	0.15
Osteoporosis diagnosis by doctor (2013–14)			
No	0.95	0.95	0.95
Yes	0.05	0.05	0.05
Osteoporosis diagnosis by doctor (2015–16)			
No	0.95	0.95	0.95
Yes	0.05	0.05	0.05

Abbreviations: GED = General Educational Development Test; SE = standard error.

**Table 2:** Descriptive Statistics for Samples in Models 5–7

Variable	Model 5 (*N* = 11,682)	Model 6 (*N* = 5,490)	Model 7 (*N* = 9,753)
	Mean (SE)/Proportion	Mean (SE)/Proportion	Mean (SE)/Proportion
Age	69.77 (0.16)	70.04 (0.21)	69.79 (0.18)
Sex and race/ethnicity identity			
Female and Black/African American	0.05	0.05	0.04
Male and Black/African American	0.03	0.01	0.02
Female and Another race/ethnicity	0.03	0.03	0.02
Male and Another race/ethnicity	0.02	0.01	0.02
Female and European American/White	0.46	0.76	0.47
Male and European American/White	0.41	0.14	0.42
Education level			
Less than high school	0.13	0.10	0.10
GED	0.04	0.04	0.04
High school grad	0.29	0.30	0.29
Some college	0.25	0.27	0.25
College and above	0.28	0.29	0.31
Allostatic load	2.22 (0.02)	2.15 (0.02)	2.19 (0.02)
Weight in kilograms	82.11 (0.26)	77.25 (0.35)	82.02 (0.28)
Thyroid disease (ever)			
No	0.96	0.94	0.96
Yes	0.04	0.06	0.04
Self‐reported health			
Excellent	0.09	0.10	0.10
Very good	0.34	0.36	0.36
Good	0.33	0.32	0.33
Fair	0.18	0.17	0.16
Poor	0.06	0.05	0.05
Maternal investment	−0.09 (0.01)	−0.10 (0.01)	−0.06 (0.01)
Respondent's mother level of education			10.57 (0.07)
Respondent's father level of education			10.20 (0.08)
Childhood socioeconomic status index			0.36 (0.02)
Osteoporosis			
No	0.91	0.82	
Yes	0.09	0.18	
DXA scan in last 2 years			
Yes	0.46		0.47
No	0.54		0.53
Wave	11.90 (0.01)	11.86 (0.01)	11.90 (0.01)
DXA scan in last 2 years by wave			
2012 – No			0.50
2012 – Yes			0.50
2014 – No			0.54
2014 – Yes			0.46
2016 – No			0.55
2016 – Yes			0.45
Osteoporosis diagnosis by wave			
2012 – No	0.84		
2012 – Yes	0.16		
2014 – No	0.95		
2014 – Yes	0.05		
2016 – No	0.95		
2016 – Yes	0.05		

Abbreviation: GED = General Educational Development Test.

The adjusted McFadden's pseudo‐*R* ^2^ for Model 1 indicated a very good fit of the model to the data, 0.19,^(^ ^50^ ^)^ and the discrimination of the model was good (C statistic = 0.791). As seen in Table 3, greater maternal investment is associated with lower odds of an osteoporosis diagnosis (odds ratio [OR] = 0.80, 95% confidence interval [CI] = 0.69, 0.92). Two of the key demographic predictors were also linked to osteoporosis diagnosis, holding all else constant, though these cannot necessarily be interpreted identically to the primary exposure of maternal investment. Identifying as female was linked to higher odds of osteoporosis diagnosis (OR = 7.22, 95% CI = 5.54, 9.40). Conversely, holding all else constant, persons identifying as Black/African American (as opposed to identifying as White/European American) (OR = 0.56, 95% CI = 0.40, 0.80) had lower odds of osteoporosis diagnosis.

**Table 3:** Odds Ratios and 95% Confidence Intervals from Logistic Regression Models 1–4

Variable	Model 1 (*N* = 11,819)	Model 2 (*N* = 10,778)		Model 3 (*N* = 10,152)		Model 4 (*N* = 11,819)		
	OR	95% CI	OR	95% CI	OR	95% CI	OR	95% CI
Intercept	0.21	[0.01–4.29]	0.17	[0.01, 3.96]	0.11	[0.004, 3.15]	0.19	[0.01, 4.36]
Maternal investment	0.80	[0.69–0.92]**	0.81	[0.69, 0.96]*	0.81	[0.68, 0.96]**	0.78	[0.66, 0.91]**
Age (natural log)	4.92	[2.57–9.40]***	5.18	[2.58, 10.40]***	5.59	[2.66, 11.76]***	5.03	[2.59, 9.80]***
Sex								
Female	7.22	[5.54–9.40]***	7.28	[5.50, 9.65]***	7.40	[5.68, 9.64]***	7.22	[5.49, 9.49]***
Race and ethnicity								
Black/African American	0.56	[0.40–0.80]**	0.59	[0.41, 0.83]**	0.58	[0.42, 0.80]**	0.57	[0.39, 0.83]**
Another race/ethnicity	0.94	[0.67–1.32]	0.93	[0.64, 1.33]	0.90	[0.57, 1.42]	0.95	[0.67, 1.36]
Education								
GED	1.56	[1.05–2.32]*	1.71	[1.08, 2.71]*	1.60	[1.02, 2.49]*	1.55	[1.03, 2.35]*
High school graduate	1.27	[0.99–1.63]	1.32	[1.001, 1.75]*	1.33	[0.98, 1.80]	1.25	[0.97, 1.61]
Some college	1.39	[1.09–1.76]*	1.44	[1.10, 1.90]*	1.31	[0.98, 1.76]	1.36	[1.06, 1.73]*
College and above	1.37	[1.09–1.73]*	1.46	[1.09, 1.96]*	1.43	[1.08, 1.90]*	1.33	[1.05, 1.70]*
Allostatic load	0.90	[0.85–0.96]***	0.91	[0.85, 0.97]**	0.93	[0.87, 0.998]*	0.90	[0.85, 0.96]**
Weight in kilograms	0.99	[0.98–0.99]***	0.99	[0.98, 0.99]***	0.98	[0.98, 0.99]***	0.99	[0.98, 0.99]***
Thyroid disease (ever)								
No	0.71	[0.54–0.93]*	0.73	[0.54, 0.98]*	0.66	[0.51, 0.86]**	0.71	[0.54, 0.93]*
Self‐reported health								
Very good	1.15	[0.85–1.56]	1.08	[0.80, 1.46]	1.14	[0.82, 1.59]	1.15	[0.84, 1.58]
Good	1.73	[1.23–2.42]**	1.67	[1.20, 2.34]**	1.72	[1.18, 2.50]**	1.73	[1.22, 2.46]**
Fair	2.52	[1.77–3.59]***	2.42	[1.66, 3.52]***	2.38	[1.59, 3.55]***	2.54	[1.76, 3.67]***
Poor	3.27	[2.22–4.84]***	3.31	[2.24, 4.91]***	3.19	[2.09, 4.87]***	3.30	[2.19, 4.96]***
Childhood socioeconomic status index							1.03	[0.94, 1.13]
Respondent's mother's level of education			0.99	[0.97, 1.02]				
Respondent's father's level of education					1.004	[0.98, 1.03]		
Wave	0.50	[0.46–0.56]***	0.50	[0.45, 0.56]***	0.51	[0.46, 0.57]***	0.50	[0.45, 0.56]***

Abbreviation: CI = confidence interval, GED = General Educational Development Test, OR = odds ratio.

* *p* < 0.05.

** *p* < 0.01.

*** *p* < 0.001.

In Model 2 (Table 3) we added mother's education. This model had a lower, but still very good, pseudo‐*R* ^2^ of 0.18 and the same C statistic. The sign and relationship of the key demographic variables in Model 1 were the same in Model 2, holding all else constant. The result for maternal investment was nearly identical to that of Model 1 (OR = 0.81, 95% CI = 0.69, 0.96). Respondent's mother's education did not significantly predict osteoporosis diagnosis (OR = 0.99, 95% CI = 0.97, 1.02).

The pseudo‐*R* ^2^ for Models 3 and 4 was almost the same as for Model 1, 0.18 and 0.19, and the C statistics are approximately the same, at 0.790 and 0.791, for each model, respectively. Like Model 2, the added exposure variables for these models were not significant. The ORs for the key demographic variables were similar to those of Model 1. For maternal investment the results remained very similar to those of Model 1; for Model 3 maternal investment was nearly identical (OR = 0.81, 95% CI = 0.68, 0.96), and for Model 4 maternal investment was very slightly attenuated (OR = 0.78, 95% CI = 0.66, 0.91).

The models testing Hypothesis 3 (Models 5–7) ranged from excellent to good, with pseudo‐*R* ^2^ of 0.2165, 0.1051, and 0.2994, respectively (Table 4). The C statistics were all similarly good, with values of 0.8302, 0.8352, and 0.8326, respectively. Models 1 and 5 were similar. Maternal investment stayed significant, though slightly attenuated (OR = 0.79, 95% CI 0.67, 0.92). Having ever had a bone density scan was significantly associated with higher odds of osteoporosis diagnosis (OR = 5.69, 95% CI 4.34, 7.45). The combined sex and race/ethnicity term showed that, compared to people identifying as female and Black/African American, increased odds of an osteoporosis diagnosis were present for persons identifying as female and White/European American (OR = 1.45, 95% CI 1.05, 2.00) and decreased odds for those identifying as male and Black/African American (OR = 0.32, 95% CI 0.15, 0.67), as well as male and White/European American (OR = 0.48, 95% CI 0.34, 0.68).

**Table 4:** Odds Ratios and 95% Confidence Intervals from Logistic Regression Models 5–7

	Model 5 (*N* = 11,682)	Model 6 (*N* = 5,490)		Model 7 (*N* = 9,753)		
Variable	OR	95% CI	OR	95% CI	OR	95% CI
Intercept	0.90	[0.03, 23.18]	19.89	[0.75, 525.30]	0.00	[0.00, 0.01]***
Maternal investment	0.79	[0.67, 0.92]**	0.80	[0.69, 0.94]**	0.93	[0.76, 1.14]
Age (log)	3.80	[1.88, 7.67]***	3.56	[1.68, 7.53]**	9.61	[4.37, 21.10]***
Sex and race/ethnicity identity						
Male and Black/African American	0.32	[0.15, 0.67]**	0.50	[0.20, 1.25]	0.16	[0.09, 0.27]***
Female and another race/ethnicity	1.38	[0.82, 2.32]	1.21	[0.73, 2.01]	1.62	[0.98, 2.68]
Male and another race/ethnicity	0.60	[0.26, 1.38]	0.76	[0.21, 2.80]	0.14	[0.07, 0.27]***
Female and White/European American	1.45	[1.05, 2.00]*	1.23	[0.87, 1.74]	2.43	[1.89, 3.12]***
Male and White/European American	0.48	[0.34, 0.69]***	0.55	[0.35, 0.85]**	0.11	[0.08, 0.15]***
Education level						
GED	1.44	[0.95, 2.18]	1.32	[0.79, 2.23]	1.62	[1.05, 2.50]*
High school graduate	1.05	[0.82, 1.36]	0.95	[0.72, 1.26]	2.01	[1.57, 2.57]***
Some college	1.06	[0.84, 1.35]	0.93	[0.71, 1.21]	2.67	[2.06, 3.47]***
College and above	0.99	[0.79, 1.25]	0.89	[0.70, 1.14]	3.31	[2.45, 4.47]***
Allostatic load	0.92	[0.87, 0.98]**	0.95	[0.90, 1.01]	0.90	[0.86, 0.95]***
Weight in kilograms	0.99	[0.98, 0.99]***	0.98	[0.97, 0.98]***	1.00	[1.00, 1.01]
Thyroid disease (ever)						
No	0.81	[0.61, 1.09]	0.78	[0.59, 1.04]	0.63	[0.39, 1.02]
Self‐reported health						
Very good	1.15	[0.84, 1.58]	1.15	[0.83, 1.61]	1.06	[0.84, 1.32]
Good	1.77	[1.23, 2.55]**	1.67	[1.14, 2.44]*	1.09	[0.86, 1.38]
Fair	2.65	[1.83, 3.83]***	2.37	[1.60, 3.51]***	1.16	[0.86, 1.55]
Poor	3.54	[2.38, 5.26]***	2.84	[1.77, 4.55]***	1.12	[0.75, 1.67]
Childhood socioeconomic status index					1.03	[0.90, 1.19]
Respondent's mother's level of education					0.99	[0.97, 1.02]
Respondent's father's level of education					1.01	[0.98, 1.04]
Had a DXA scan in the last 2 years						
Yes	5.69	[4.35, 7.45]***				
Wave	0.50	[0.45, 0.55]***	0.49	[0.44, 0.54]***	0.92	[0.86, 0.98]**

Abbreviation: CI = confidence interval, GED = General Educational Development Test, OR = odds ratio.

* *p* < 0.05.

** *p* < 0.01.

*** *p* < 0.001.

Model 6, estimated for the sample of individuals who reported having a bone density scan, showed some attenuation of results from Models 1 and 5. Compared to people identifying as female and Black/African American, decreased odds of an osteoporosis diagnosis were shown for those identifying as male and White/European American (OR = 0.55, 95% CI 0.35, 0.85). No other combined sex and race/ethnicity terms were statistically significant. Maternal investment remained very similar to that of Model 5 (OR = 0.80, 95% CI 0.69, 0.93).

In Model 7 the outcome was reporting receipt of a bone density scan. None of the childhood background predictors was a significant factor for obtaining a bone density scan. By contrast, the most important predictors were demographic. Compared to persons identifying as female and Black/African American, those identifying as female and White/European American had much higher odds of reporting a bone density scan (OR = 2.43, 95% CI 1.89, 3.12), while those identifying as male and Black/African American (OR = 0.16, 95% CI 0.09, 0.27), another race/ethnicity (OR = 0.14, 95% CI 0.07, 0.27), or White/European American (OR = 0.11, 95% CI 0.08, 0.15) all experienced much lower odds.

## Discussion

Building on the literature on the long arm of childhood, we tested three hypotheses relating to increased risk of osteoporosis diagnosis and childhood SES, maternal investment, and underdiagnosis in persons of color. We found no evidence to support Hypothesis 1 that lower childhood SES was associated with a higher risk of osteoporosis diagnosis in adulthood. Rather, maternal investment, a measure of quality of relationship with the mother that may serve as a proxy for maternal social capital, was the only childhood predictor that related to odds of osteoporosis. This finding that supports Hypothesis 2, which stated that separately from childhood SES, lower maternal investment links to a higher risk of osteoporosis diagnosis. As expected, models show some evidence of underdiagnosis of persons identifying as Black/African American (as compared to those identifying as White/European American), supporting Hypothesis 3, which stated that there would be evidence of underdiagnosis among persons identifying with non‐White racial/ethnic groups. This was seen primarily for females. Because this sample is from a nationally representative study, these findings are generalizable across middle‐ to older‐age U.S. adults.

Given the extensive literature on the long arm of childhood that focuses primarily on SES, it was somewhat surprising that we did not see evidence of childhood SES affecting getting an osteoporosis diagnosis, although our models included education, a potential mediator of this relationship. Yet we are not alone in finding no connection between childhood SES and later‐life bone health.^(^ ^8^ ^)^ Additionally, although childhood SES could be important for bone health in earlier life through its influence on adulthood SES or through nutritional factors as previously posited, there is a long intervening period in which adults are making choices about health‐harming behaviors and experiencing other kinds of exposures, all of which may attenuate any relationship between childhood SES and osteoporosis diagnosis.^(^ ^51^ ^)^

The results for maternal investment were as hypothesized, though maternal investment in the sense it was conceptualized here is not widely discussed as a predictor of later‐life health. However, prior research demonstrated that greater parental investment during childhood could be beneficial for outcomes in adulthood. Higher‐quality mother–child and father–son relationships are associated with lower psychological stress and emotional reactivity, respectively, in adulthood.^(^ ^52^ ^)^ Higher‐quality familial relationships relate to higher psychological well‐being of emerging adults.^(^ ^53^ ^)^ Moreover, recent studies linked greater maternal investment to better physical and/or mental health.^(^ ^16^, ^22^ ^)^ This suggests that continuous maternal and paternal investment can be crucial to later‐life health outcomes and supports our findings with maternal investment.

We posited the potential role of nutritional deficiencies is the connection between childhood exposures and osteoporosis diagnosis in adulthood. Although we do not see evidence supporting a relationship between childhood SES and osteoporosis diagnosis, it is plausible the maternal investment variable captures some aspects of mothers' support for children's nutrition. Society expects good mothers to keep their children fed and clothed,^(^ ^54^ ^)^ and though respondents in the HRS were children from roughly the late 1930s through the early 1960s, their mothers likely felt similar pressures and sought to provide good nutrition to their children. Although times have changed, recent research supports the notion that mothers play an important role for children's nutrition and possibly later bone health; mothers that received education on osteoporosis risk factors reported changes in their child's calcium intake and physical activity.^(^ ^15^ ^)^ Thus, maternal investment could be capturing aspects of both theoretical pathways discussed between childhood exposures and later‐life health (Figure 1): the pathway to adult SES and the nutrition pathway.

Our results agree with many studies over the past 20 years^(^ ^31^ ^)^: there is underdiagnosis of osteoporosis among people identifying as Black/African American. However, the models incorporating the intersectional sex and race/ethnicity combination measures provide important nuance. The largest racial/ethnic disparity appears to be occurring between individuals identifying as female and White/European American compared to individuals identifying as female and Black/African American. For those identifying as male, the CIs across race/ethnicity are overlapping. In prior research the lower odds of an osteoporosis diagnosis for persons identifying as Black/African American were fully attenuated once access to a bone density scan was controlled,^(^ ^33^ ^)^ supporting findings that practitioners may use race/ethnicity to decide on preventive screenings for persons identifying as Black/African American, even though it is not a relevant risk factor,^(^ ^32^, ^55^ ^)^ because it is well established that individuals identifying as Black/African American women, for example, have similarly substantial rates of low bone density to persons identifying as White/European American women.^(^ ^32^ ^)^ Inequities for people identifying as Black/African American women, as opposed to White/European American women, extend to care,^(^ ^55^, ^56^ ^)^ treatment,^(^ ^55^ ^)^ and postfracture referrals.^(^ ^56^ ^)^ Thus, the lower odds of an osteoporosis diagnosis estimated in this study for people identifying as Black/African American are probably a result of underdiagnosis, as supported by Model 7's results regarding receipt of a bone density scan. Our results suggest the possibility of underdiagnosis for those identifying as male (regardless of race/ethnicity) and those identifying as female and Black/African American. Systemic racism in the health care system perpetuates unequal care and access to treatment and tests for diseases, including osteoporosis, which puts persons of color at a disadvantage.^(^ ^57^ ^)^ For osteoporosis specifically, screening primarily targets persons who identify as non‐Hispanic White (and female), with screening disparities negatively affecting people who identify as non‐Hispanic Black, Hispanic or Latina/o, and Asian living in the United States.^(^ ^58^ ^)^ This phenomenon necessitates targeted clinician initial and continuing education that includes DEI training for reflection and adjustment of providing health care to persons of color and those historically viewed as less vulnerable to osteoporosis. DEI training can further help clinicians to appropriately support persons from low SES in accessing the medical care they need.

### Limitations

This study had biases, including an overly simplified race/ethnicity variable due to sample size. The sex variable was binary, which did not reflect its underlying biological continuum. Further, the childhood variables were developed from answers to questions asking respondents to reflect back to age 16,^(^ ^16^ ^)^ which may be subject to recall bias. For example, for most respondents their mothers were last responsible for their well‐being 40+ years ago, which is a long time for alternative influences to have an effect on later‐life health and memories of their mother. Recall bias can be further extended to other questions, such as self‐reported health. Additionally, respondents were not asked about nutrition during childhood, and the sample was not studied during early life, so was is not possible to definitively address whether maternal investment was related to osteoporosis diagnosis through adult SES, nutritional factors during childhood, or other pathways. This study is an analysis of association and does not allow us to draw causal inferences from the results, though this is an important direction for future research.

## Conclusions

The results of this study indicate that the long arm of childhood extends to osteoporosis, a common and costly progressive condition, though not through childhood SES as much as through maternal investment. As younger cohort studies reach middle age, it may be possible to further parse the mechanisms through which investment is beneficial. Additionally, future research should continue this line of inquiry, applying sociological and anthropological theory, to examine how social factors, including early‐life circumstances, can influence the development of disease, receipt of diagnoses, and experience of the health care system.

## Author Contributions

**Margaret Gough Courtney:** Conceptualization; formal analysis; funding acquisition; methodology; project administration; supervision; writing – original draft; writing – review and editing. **Josephine Roberts:** Conceptualization; formal analysis; project administration; writing – original draft; writing – review and editing. **Yadira Quintero:** Conceptualization; formal analysis; project administration; writing – original draft; writing – review and editing. **K. Godde:** Conceptualization; formal analysis; funding acquisition; methodology; project administration; supervision; writing – original draft; writing – review and editing.

## References

[1] Haas S . Trajectories of functional health: the ‘long arm’ of childhood health and socioeconomic factors. Soc Sci Med. 2008;66(4):849–861. 10.1016/j.socscimed.2007.11.004. 18158208

[2] Hayward MD , Gorman BK . The long arm of childhood: the influence of early‐life social conditions on men's mortality. Demography. 2004;41(1):87–107. 10.1353/dem.2004.0005. 15074126

[3] Vable AM , Gilsanz P , Kawachi I . Is it possible to overcome the ‘long arm’ of childhood socioeconomic disadvantage through upward socioeconomic mobility? J Public Health. 2019;41(3):566–574. 10.1093/pubmed/fdz018. PMC7967879 30811528

[4] Juhola J , Oikonen M , Magnussen CG , et al. Childhood physical, environmental, and genetic predictors of adult hypertension. Circulation. 2012;126(4):402–409. 10.1161/CIRCULATIONAHA.111.085977. 22718800

[5] Lewiecki EM , Ortendahl JD , Vanderpuye‐Orgle J , et al. Healthcare policy changes in osteoporosis can improve outcomes and reduce costs in the United States. JBMR Plus. 2019;3(9):1–7. 10.1002/jbm4.10192. PMC6808223 31667450

[6] Wright NC , Looker AC , Saag KG , et al. The recent prevalence of osteoporosis and low bone mass in the United States based on bone mineral density at the femoral neck or lumbar spine. J Bone Miner Res. 2014;29(11):2520–2526. 10.1002/jbmr.2269. 24771492 PMC4757905

[7] Wilson S , Sharp CA , Davie MWJ . Health‐related quality of life in patients with osteoporosis in the absence of vertebral fracture: a systematic review. Osteoporos Int. 2012;23(12):2749–2768. 10.1007/s00198-012-2050-6. 22814944

[8] Crandall C , Merkin SS , Seeman TE , Greendale GA , Binkley N , Karlamangla AS . Socioeconomic status over the life‐course and adult bone mineral density: the Midlife in the U.S Study. Bone. 2012;51(1):107–113. 10.1016/j.bone.2012.04.009. 22543227 PMC3371160

[9] Karlamangla AS , Mori T , Merkin SS , et al. Childhood socioeconomic status and adult femoral neck bone strength: findings from the midlife in the United States study. Bone. 2013;56(2):320–326. 10.1016/j.bone.2013.06.021. 23810840 PMC3784306

[10] Ross CE , Mirowsky J . The interaction of personal and parental education on health. Soc Sci Med. 2011;72(4):591–599. 10.1016/j.socscimed.2010.11.028. 21227556 PMC3049298

[11] Link BG , Phelan J . Social conditions as dundamental causes of disease. J Health Soc Behav. 1995;35:80. 10.2307/2626958. 7560851

[12] Alaimo K , Olson CM , Frongillo EA , Briefel RR . Food insufficiency, family income, and health in US preschool and school‐aged children. Am J Public Health. 2001;91(5):781–786 https://www.ncbi.nlm.nih.gov/pmc/articles/PMC1446676/. 11344887 10.2105/ajph.91.5.781 PMC1446676

[13] Cooper C , Westlake S , Harvey N , Javaid K , Dennison E , Hanson M . Review: developmental origins of osteoporotic fracture. Osteoporos Int. 2006;17(3):337–347. 10.1007/s00198-005-2039-5. 16331359

[14] Skinner MF , Hung JTW . Social and biological correlates of localized enamel hypoplasia of the human deciduous canine tooth. Am J Phys Anthropol. 1989;79(2):159–175. 10.1002/ajpa.1330790204. 2742003

[15] Winzenberg TM , Oldenburg B , Frendin S , De Wit L , Jones G . A mother‐based intervention trial for osteoporosis prevention in children. Prev Med. 2006;42(1):21–26. 10.1016/j.ypmed.2005.11.006. 16336993

[16] Vable AM , Gilsanz P , Nguyen TT , Kawachi I , Glymour MM . Validation of a theoretically motivated approach to measuring childhood socioeconomic circumstances in the health and retirement study. PLoS One. 2017;12(10):1–23. 10.1371/journal.pone.0185898. PMC5640422 29028834

[17] Dufur MJ , Parcel TL , Troutman KP . Does capital at home matter more than capital at school? Social capital effects on academic achievement. Res Soc Stratification Mobility. 2013;31:1–21. 10.1016/j.rssm.2012.08.002.

[18] von Otter C , Stenberg S‐Å . Social capital, human capital and parent–child relation quality: interacting for children's educational achievement? Br J Sociol Educ. 2015;36(7):996–1016. 10.1080/01425692.2014.883275.

[19] De Silva MJ , Harpham T . Maternal social capital and child nutritional status in four developing countries. Health Place. 2007;13(2):341–355. 10.1016/j.healthplace.2006.02.005. 16621665

[20] Harpham T , De Silva MJ , Tuan T . Maternal social capital and child health in Vietnam. J Epidemiol Community Health. 2006;60(10):865–871. 10.1136/jech.2005.044883. 16973533 PMC2566054

[21] Gundersen C , Lohman BJ , Garasky S , Stewart S , Eisenmann J . Food security, maternal stressors, and overweight among low‐income US children: results from the National Health and Nutrition Examination Survey (1999–2002). Pediatrics. 2008;122(3):e529–e540. 10.1542/peds.2008-0556. 18762488

[22] Gough Courtney M , Quintero Y , Godde K . Assessing the roles of demographic, social, economic, environmental, health‐related, and political factors on risk of osteoporosis diagnosis among older adults. Arch Osteoporos. 2021;16(1):1–12. 10.1007/s11657-021-01042-0. 34817704 PMC8722370

[23] Brady D , Guerra C , Kohler U , Link B . The long arm of prospective childhood income for mature adult health in the United States. J Health Soc Behav. 2022;63(4):543–559. 10.1177/00221465221081094. 35253530 PMC10510903

[24] Sarafrazi N , Wambogo EA , Shepherd JA . Osteoporosis or low bone mass in older adults: United States, 2017‐2018. NCHS Data Brief. 2021;405:1–8. 34029181

[25] Becker DJ , Kilgore ML , Morrisey MA . The societal burden of osteoporosis. Curr Rheumatol Rep. 2010;12(3):186–191. 10.1007/s11926-010-0097-y. 20425518

[26] Bentler SE , Liu L , Obrizan M , et al. The aftermath of hip fracture: discharge placement, functional status change, and mortality. Am J Epidemiol. 2009;170(10):1290–1299. 10.1093/aje/kwp266. 19808632 PMC2781759

[27] Gillespie CW , Morin PE . Osteoporosis‐related health services utilization following first hip fracture among a cohort of privately‐insured women in the United States, 2008‐2014: an observational study. J Bone Miner Res. 2017;32(5):1052–1061. 10.1002/jbmr.3079. 28229485

[28] Haas SA . Health selection and the process of social stratification: the effect of childhood health on socioeconomic attainment. J Health Soc Behav. 2006;47(4):339–354. 10.1177/002214650604700403. 17240924

[29] Phelan JC , Link BG , Tehranifar P . Social conditions as fundamental causes of health inequalities: theory, evidence, and policy complications. J Health Soc Behav. 2010;51(1_suppl):S28–S40. 10.1177/0022146510383498. 20943581

[30] Phelan JC , Link BG . Is racism a fundamental cause of inequalities in health? Annu Rev Sociol. 2015;41(1):311–330. 10.1146/annurev-soc-073014-112305.

[31] Burgess DJ , Fu SS , van Ryn M . Why do providers contribute to disparities and what can be done about it? J Gen Intern Med. 2004;19(11):1154–1159. 10.1111/j.1525-1497.2004.30227.x. 15566446 PMC1494785

[32] Wilkins CH , Goldfeder JS . Osteoporosis screening is unjustifiably low in older African‐American women. J Natl Med Assoc. 2004;96(4):461–467 https://www.ncbi.nlm.nih.gov/pmc/articles/PMC2595016/. 15101666 PMC2595016

[33] Godde K , Gough Courtney M , Roberts J . Health insurance coverage as a social determinant of osteoporosis diagnosis in a population‐based cohort study of older American adults. J Appl Gerontol. 2022;42(2):302–312. 10.1177/07334648221132792. 36222070 PMC9841821

[34] Health and Retirement Study . Health and Retirement Study, (Core Data) Public Use Dataset. Produced and Distributed by the University of Michigan with Funding from the National Institute on Aging (Grant Number NIA U01AG009740). Ann Arbor, MI: University of Michigan; 2020b.

[35] Health and Retirement Study . (2017c). Sample sizes and response rates. https://hrs.isr.umich.edu/sites/default/files/biblio/ResponseRates_2017.pdf

[36] Sonnega A , Faul JD , Ofstedal MB , Langa KM , Phillips JW , Weir DR . Cohort profile: the health and retirement study (HRS). Int J Epidemiol. 2014;43(2):576–585. 10.1093/ije/dyu067. 24671021 PMC3997380

[37] Health and Retirement Study . *RAND HRS Longitudinal File 2016* (*V2*). Produced by the RAND Center for the Study of Aging, with Funding from the National Institute on Aging and the Social Security Administration. Santa Monica, CA: University of Michigan; 2020c.

[38] Health and Retirement Study . Cross‐Wave Race and Ethnicity File. Produced and Distributed by the University of Michigan with Funding from the National Institute on Aging (Grant Number NIA U01AG009740). Ann Arbor: MI; 2014 https://hrsdata.isr.umich.edu/data-products/2008-telomere-data.

[39] Health and Retirement Study . 2015 Fall Life History Mail Survey Occupation and Industry Data | Public Use Dataset. Produced and Distributed by the University of Michigan with Funding from the National Institute on Aging (Grant Number NIA U01AG009740). Ann Arbor: MI; 2015 https://hrs.isr.umich.edu/news/2015-life-history-mail-survey-lhms.

[40] Health and Retirement Study . 2017 Fall Life History Mail Survey Occupation and Industry Data | Public Use Dataset. Produced and Distributed by the University of Michigan with Funding from the National Institute on Aging (Grant Number NIA U01AG009740). Ann Arbor: MI; 2017a https://hrs.isr.umich.edu/data-products/restricted-data/available-products/11273.

[41] Health and Retirement Study . *Biomarker Data 2006–2016*. Produced and Distributed by the University of Michigan with Funding from the National Institute on Aging (Grant Number NIA U01AG009740). Ann Arbor, MI: University of Michigan; 2020a.

[42] Health and Retirement Study . (2017b). Institutional review board information. https://hrs.isr.umich.edu/sites/default/files/biblio/HRS_IRB_Information-10-2017.pdf

[43] Hernán MA . The C‐word: scientific euphemisms do not improve causal inference from observational data. Am J Public Health. 2018;108(5):616–619. 10.2105/AJPH.2018.304337. 29565659 PMC5888052

[44] Maldonado G , Greenland S . Simulation study of confounder‐selection strategies. Am J Epidemiol. 1993;138(11):923–936. 10.1093/oxfordjournals.aje.a116813. 8256780

[45] McCrory C , Fiorito G , Ni Cheallaigh C , et al. How does socio‐economic position (SEP) get biologically embedded? A comparison of allostatic load and the epigenetic clock(s). Psychoneuroendocrinology. 2019;104(2019):64–73. 10.1016/j.psyneuen.2019.02.018. 30818253

[46] Barr DA . Health Disparities in the United States. 2nd ed. Johns Hopkins University Press; 2014.

[47] Ginaldi L , Di Benedetto MC , De Martinis M . Osteoporosis, inflammation and ageing. Immun Ageing. 2005;2(1):14. 10.1186/1742-4933-2-14. 16271143 PMC1308846

[48] Lumley T . Analysis of complex survey samples. J Stat Softw. 2004;9(8):1–19. 10.18637/jss.v009.i08.

[49] R Core Team . R: A Language and Environment for Statistical Computing. R Foundation for Statistical Computing; 2020. http://www.R-project.org

[50] McFadden D . *Quantitative methods for analysing travel behaviour of individuals* (Cowles Foundation discussion paper no. 474). New Haven: Yale University; 1977.

[51] Brennan SL , Pasco JA , Urquhart DM , Oldenburg B , Hanna F , Wluka AE . Educational achievement and fracture risk: response to Clark and Tobias. Osteoporos Int. 2010;21(9):1623. 10.1007/s00198-009-1112-x. 20012019

[52] Mallers MH , Neupert SD , Charles ST , Almeida DM , Mallers MH , Psychology DO . Perceptions of childhood relationships with mother and father: daily emotional and stressor experiences in adulthood. Am Psychol Assoc. 2010;46(6):1651–1661. 10.1037/a0021020 PMC3468907 20873925

[53] García Mendoza MDC , Sánchez Queija I , Parra Jiménez Á . The role of parents in emerging Adults' psychological well‐being: a Person‐oriented approach. Fam Process. 2019;58(4):954–971. 10.1111/famp.12388. 30198562

[54] Devault ML . Feeding the family: The Social Organization of Caring as Gendered Work. University of Chicago Press, 1994.

[55] Miller RG , Ashar BH , Cohen J , et al. Disparities in osteoporosis screening between at‐risk African‐American and white women. J Gen Intern Med. 2005;20(9):847–851. 10.1111/j.1525-1497.2005.0157.x. 16117754 PMC1490213

[56] Mudano AS , Casebeer L , Patino F , et al. Racial disparities in osteoporosis prevention in a managed care population. South Med J. 2003;96(5):445–451. 10.1097/01.SMJ.0000053918.93363.B0. 12911182

[57] Dhaliwal R , Pereira RI , Diaz‐Thomas AM , Powe CE , Yanes Cardozo LL , Joseph JJ . Eradicating racism: an Endocrine Society policy perspective. J Clin Endocrinol Metabol. 2022;107(5):1205–1215. 10.1210/clinem/dgab896. 35026013

[58] Noel SE , Santos MP , Wright NC . Racial and ethnic disparities in bone health and outcomes in the United States. J Bone Miner Res. 2021;36(10):1881–1905. 10.1002/jbmr.4417. 34338355 PMC8607440

